# U.S. Emergency Department Visits by Persons With Dementia: Impact of Medicare Claims Data and Undiagnosed Dementia

**DOI:** 10.1111/acem.70236

**Published:** 2026-02-02

**Authors:** Alexander X. Lo, Michael Crowe, Richard E. Kennedy

**Affiliations:** ^1^ Department of Emergency Medicine Northwestern University Feinberg School of Medicine Chicago Illinois USA; ^2^ Center for Health Services & Outcomes Research Northwestern University Feinberg School of Medicine Chicago Illinois USA; ^3^ Department of Psychology University of Alabama at Birmingham (UAB) Birmingham Alabama USA; ^4^ Division of Gerontology, Geriatrics, and Palliative Care, Department of Medicine University of Alabama at Birmingham (UAB) Birmingham Alabama USA

**Keywords:** dementia, emergency department, epidemiology

The emergency department (ED) has arguably become the most important nexus for healthcare for persons living with dementia (PLWD), serving as the default care site for PLWD with unmet care needs and poor access to care [1]. The ED is also the predominant entry portal to the hospital, accounting for up to 70% of admissions for older persons [2]. Half of the current ~7.2 million PLWD in the U.S. have one or more ED visits per year, and 37% of PLWD visiting the ED are hospitalized [3].

The challenges and limitations of ED care for PLWD have been emphasized at the national level. Notably, a 2022 National Institute on Aging‐funded interdisciplinary working group and consensus conferences highlighted key evidence gaps for PLWD in the ED that must be addressed before strategies or policies for best ED practices could be developed [4]. Ultimately, a priority question arose: How can we best identify and enumerate the population of PLWD seeking care in the ED? An equally important question is how can we best identify PLWD who do not have a formal diagnosis of dementia?

Galske and colleagues examined ED visit characteristics associated with PLWD using 2019 Medicare Part B claims data and found that an overall median estimate of 15.6% of ED visits across 3178 EDs in 2019 were by Medicare beneficiaries with a documented diagnosis of dementia. This estimated proportion varied with increasing age intervals, i.e., 6.6% for ages 65–74 years, 17.8% for ages 75–84 years, and 32.2% for age 85 years and older, as well as by insurance status (30.6% for beneficiaries with dual eligibility vs. 12.8% for those without) and teaching hospital status (14.8% for teaching hospitals vs. 15.7% for non‐teaching hospitals) [5]. Given the current scant evidence on ED utilization by PLWD, this study represents a useful advance in our understanding of this topic, particularly with the focus on ED patients aged 85 years and older, for whom there is even less evidence. However, the methodological limitations inherent in this study are also noteworthy and highlight the challenges and complexities of using Medicare claims data.

There are several potential ways of identifying dementia among ED patients. First would be by self‐report, but PLWD may not have been diagnosed or may be unable to report the diagnosis, and caregivers may be unaware of the dementia. Second would be by cognitive screening and testing, but this is difficult and logistically prohibitive to do in the ED. So researchers may choose to restrict their scope of inquiry only to individuals with a formal diagnosis of dementia and rely on administrative data, which has several strengths but also weaknesses. Medicare claims data allow a national examination of PLWD and their ED utilization, capturing visits by participants across multiple health systems, but with several associated limitations. There is not a consistent definition of dementia using claims data. Different dementia case definition algorithms based on billing codes have been described, with each algorithm employing varying periods of consecutive Medicare claims data (i.e., “look‐back periods”), most commonly either a 1‐ or 3‐year look‐back window as part of the search algorithms [6].

A frequently used case definition algorithm is the Centers for Medicare and Medicaid Services (CMS) Chronic Conditions Warehouse (CCW) algorithm with a 3‐year look‐back [7]. A strength of the CCW algorithm is its high sensitivity with its requirement of only one claim across the 3 years. While this algorithm may reduce the potential under‐diagnosis of dementia, it sacrifices specificity, and the longer claim period can have practical drawbacks in research, e.g., in prospective observation studies or pragmatic trials [8]. The Bynum algorithm utilizes a 1‐year look‐back, which offers pragmatic advantages over the CCW algorithm and with reasonable accuracy in terms of a higher specificity and positive predictive value, but with lower sensitivity [8]. Other algorithms incorporate Medicare Part D data to identify prescriptions for medications as a means to increase the sensitivity of their case definition [7].

Studies examining various algorithms found that a 3‐year look‐back window demonstrated better accuracy overall than a 1‐year look‐back window [6]. In the case of the Bynum algorithm, the 3‐year (compared with a 1‐year) look‐back showed higher sensitivity (79% vs. 64%) and lower specificity (88% vs. 93%) but no significant differences in positive (50% vs. 58%) and negative (97% vs. 95%) predictive values [6].

No matter which Medicare claims algorithm or look‐back period is utilized, studies have consistently found that several subgroups of PLWD are missed by Medicare claims data, notably racial minorities and particularly African Americans [6, 8]. Claims data were also more likely to identify individuals with characteristics indicating more severe dementia, e.g., lower cognitive test scores and greater functional limitations [8].

Studies utilizing Medicare claims have typically only included data from Fee for Service (FFS) Medicare parts A and B. While part B captures ED visits as outpatient encounters, this only applies to ED visits for patients who are not admitted to the hospital. ED visits resulting in admission to hospital are typically captured by Medicare Part A as part of the inpatient claim. Given the higher propensity of hospital admissions among older ED patients, especially those with complex care needs or multi‐morbidity [9], Part A would arguably account for the majority of older persons seeking care in the ED. The inclusion of Medicare part C, which represents Medicare Advantage beneficiaries, is less typical but is becoming more common among PLWD subscribing to Medicare Advantage plans relative to traditional FFS Medicare plans [7].

The Galske study applied the Bynum algorithm with a 1‐year look‐back but relied only on 2019 Medicare Part B claims data [5]. Despite the limitations of using only Part B claims and those intrinsic to the algorithm employed, as acknowledged by the authors, their report, nonetheless, contributes useful knowledge to the evidence gap of PLWD in the ED. The absence of Part A claims does not necessarily undermine the findings, given that PLWD who present to the ED and are hospitalized are substantially different from those who visit the ED and are discharged home, with distinct differences in demographic, social, and clinical characteristics [10]. Future studies characterizing ED utilization by persons with diagnosed dementia should consider applying the Bynum algorithm with a 3‐year look‐back window on claims data comprising Medicare FFS Parts A and B, as well as Part C, in order to obtain a more comprehensive picture and add to the nascent evidence base. With the complexities and idiosyncrasies of each of the distinct data sources, ascertainment algorithms and temporal windows, research methodologies, and the challenge of finding undiagnosed dementia cases, there is no single perfect approach to ascertaining, enumerating, and characterizing all ED visits by PLWD in the U.S. However, in a vacuum of knowledge, even new data in a narrow scope still can be important stepping stones for further discovery, provided the strengths and limitations of the methods are transparent.

For ED‐based research on dementia, the impact of not capturing PLWD without a formal diagnosis of dementia can be significant. Up to 60% of PLWD do not have a formal diagnosis of dementia, and these persons are more likely to be older, male, unmarried, of Hispanic origin, with fewer years of education, with less severe cognitive or functional impairments, more likely to seek ED care by themselves, and be hospitalized [11].

Thus, studies of PLWD utilizing Medicare claims data for diagnosis may not generalize well to a significant proportion of this population. Furthermore, the issues with claims‐based diagnoses are not restricted to the research setting. In clinical practice, poor documentation of dementia would lead to inaccurate information on the prevalence and cost of dementia for payor organizations, with potential negative impacts on development and funding of programs for identification and treatment of patients with these disorders. At the individual level, poor documentation could lead to a lack of services for PLWD as analysis of administrative data is a common case finding method among managed care organizations. Poor documentation of dementia, particularly prior to the more advanced stages, would also hinder insurance approval for medications that are indicated for specific dementia subtypes and in the early stage of the disease.

The challenge of identifying undiagnosed dementia in the ED is underscored by the findings of the GEAR 2.0 ADC working group of the lack of evidence on standardized tools and methodologies to screen for dementia in the ED [4]. Having a proven ED screen for dementia would serve as a critical step toward the development of a robust approach to capturing all PLWD in the ED and especially those who have not yet been formally diagnosed. Nonetheless, the ED will continue to serve its critical role in the care of PLWD, whether with a formal diagnosis or not. As ED services for PLWD continue to evolve, changes in practice, protocol, and policies must be driven by data that accurately represents the entirety of the patient population.

## Author Contributions

A.X.L. and R.E.K. are responsible for the concept and contents of this manuscript. All authors were involved in the writing and editing of this manuscript.

## Funding

This work was supported by National Institute on Aging, RF1AG096921, P30AG086401, R01AG06476.

## Linked Article

Visit Characteristics of Emergency Departments Caring for Persons Living With Dementia: A Nationally Representative Sample. https://doi.org/10.1111/acem.70160.

## Data Availability

Data sharing not applicable to this article as no datasets were generated or analysed during the current study.
